# Comparison of X-ray-Mammography and Planar UWB Microwave Imaging of the Breast: First Results from a Patient Study

**DOI:** 10.3390/diagnostics8030054

**Published:** 2018-08-21

**Authors:** Dennis Wörtge, Jochen Moll, Viktor Krozer, Babak Bazrafshan, Frank Hübner, Clara Park, Thomas J. Vogl

**Affiliations:** 1 Department of Physics, Goethe University of Frankfurt, Max-von-Laue-Str. 1, 60438 Frankfurt am Main, Germany; dennis.woertge@stud.uni-frankfurt.de (D.W.); krozer@physik.uni-frankfurt.de (V.K.); 2 Institute for Diagnostic and Interventional Radiology, Goethe University Hospital Frankfurt, Theodor-Stern-Kai 7, 60590 Frankfurt am Main, Germany; Babak.Bazrafshan@kgu.de (B.B.); Frank.Huebner@kgu.de (F.H.); Clara.Park@kgu.de (C.P.); t.vogl@em.uni-frankfurt.de (T.J.V.)

**Keywords:** microwave breast imaging, UWB diagnostics, patient study

## Abstract

Hemispherical and cylindrical antenna arrays are widely used in radar-based and tomography-based microwave breast imaging systems. Based on the dielectric contrast between healthy and malignant tissue, a three-dimensional image could be formed to locate the tumor. However, conventional X-ray mammography as the golden standard in breast cancer screening produces two-dimensional breast images so that a comparison between the 3D microwave image and the 2D mammogram could be difficult. In this paper, we present the design and realisation of a UWB breast imaging prototype for the frequency band from 1 to 9 GHz. We present a refined system design in light of the clinical usage by means of a planar scanning and compare microwave images with those obtained by X-ray mammography. Microwave transmission measurements were processed to create a two-dimensional image of the breast that can be compared directly with a two-dimensional mammogram. Preliminary results from a patient study are presented and discussed showing the ability of the proposed system to locate the tumor.

## 1. Introduction

Breast cancer had the highest mortality in women in the European Union towards the end of the 1990s and the beginning of the 2000s, and a steady decrease in mortality is reported by Malvezzi et al. [1]. This trend could be explained with the implementation of screening programs that enable early breast cancer detection and treatment. However, according to the same authors, breast cancer still has the second highest predicted mortality rate in women with about 92,700 deaths in the European Union in 2018. These numbers show the need to develop and improve medical diagnosis techniques for the detection of breast cancer.

Microwave techniques have potential importance for medical diagnosis given by complementary diagnostic information about breast tissues compared to established techniques such as X-ray, ultrasound or MRI [2,3,4,5]. A classification of the available prototype systems for microwave-based diagnostics can be made in terms of the antenna array arrangement that can be either three-dimensional (hemisperical or cylindrical) or two-dimensional. In most of the available three-dimensional microwave imaging systems, the patient lies in a prone position on an examination table with the breast immersed in a hemispherical cup or cylindrical tank. Several three-dimensional prototype systems should be briefly introduced: a multi-static radar-based breast imaging prototype operates in the 3 to 8 GHz band and is validated in a patient study [6]. Flexible antenna arrays for microwave breast imaging are demonstrated in [7,8]. Further multistatic prototype systems are reported in Helbig et al. [9] and Yang et al. [10]. Song et al. [11] propose a hand-held impulse radar and report successful application in breast phantoms and patients. A monostatic breast imaging system is proposed in [12,13], which adaptively conforms to the breast’s shape by means of a laser positioning system. Besides the radar-based three-dimensional imaging systems, a number of tomography-based microwave breast imaging systems can be found in the literature—proposed, for example, by Zhurbenko et al. [14] and Rydholm et al. [15].

Only a few two-dimensional imaging systems for breast cancer detection are reported so far. Tajik et al. [16] performed a two-dimensional scanning of a compressed breast phantom and show quantitative microwave holography imaging in real time in the frequency band from 3 to 8 GHz. A UWB system for estimating the bulk dielectric properties of breast tissues is demonstrated in [17] for the frequency band from 1.5 to 10 GHz. The system consists of five transmitting and receiving microwave antennas on top and below the breast. Although the latter system accounts for different breast shapes and sizes by means of breast compression, limitations are given by only a few measurement positions.

O’Loughlin et al. [18] studied the currently available microwave breast imaging systems and came to the conclusion that operational microwave imaging systems have to address the following challenges to clinical practice:

- developing quality systems to ensure repeatability and safety,
- designing sufficiently powered, large-scale clinical trials to address sensitivity and specificity,
- identifying how microwave imaging can have a positive impact in the current patient pathway,
- refining system design in light of clinical usage.

The proposed microwave imaging (MWI) in this paper contributes to the last two items. A positive impact of microwave imaging is demonstrated here by comparing X-ray images to microwave images in a patient study to show the value of microwave imaging. This potential additional diagnostic information may lead to an improvement in the current patient pathway. Moreover, the proposed MWI method is not harmful for the patient because non-ionizing radiation is used. A refined system design is achieved by compressing the breast, similar to mammography, to guarantee a good mechanical contact and to account for different breast shapes and sizes in a simple but effective way. On top of that, the UWB antennas are designed in light of the permittivity of the skin so that a coupling medium that potentially increases measurement uncertainty can be avoided.

The novelty of the proposed prototype system in relation to those breast imaging systems described in literature is given in the way the diagnostic image is computed. In contrast to other image reconstruction techniques, the amount of signal attenuation could be exploited, which is higher in malignant than healthy tissue [19]. A root-mean-square (RMS) analysis of the transmitted UWB radar pulses is calculated that does not require information about the wave speed, which is challenging in every digital beamformer approach. Moreover, this indicator is used to produce two-dimensional projection from a three-dimensional breast, similar to X-ray mammography. In addition, the proposed methodology does not need iterative computations, which is beneficial compared to image formation in tomography systems. The image reconstruction method described in this paper is simple and effective, and provides real-time capabilities.

## 2. Materials and Methods

### 2.1. Ethical Approval

For the experiments involving human participants in this work, an ethical approval with the reference number 2/16 was obtained from the ethics committee of the J. W. Goethe-Universitätsklinikum. The ethical approval was issued at 6 April 2016 and is valid for 3 years.

### 2.2. Experimental Setup

**Figure 1:**
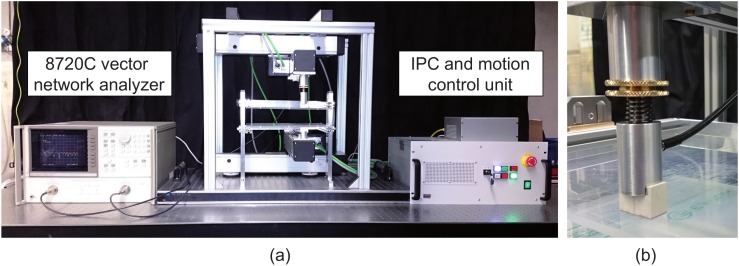
(**a**) Experimental setup, after [21]; (**b**) spring-based mechanism for vertical adjustment of the top antenna to provide a good mechanical coupling even in the case of variable breast sizes and shapes.

Figure 1a shows a photo of the microwave breast imaging system where the breast is compressed by two 5 mm thick plexiglass plates with a low loss at microwave frequencies. While the lower plate had a fixed position, the upper plate was adjustable in vertical direction to account for different breast shapes and sizes as depicted in Figure 1b. The setup consists of two UWB bowtie antennas [20] that are connected to a 8720C vector network analyzer (Keysight Technologies Deutschland GmbH, Böblingen, Germany) using flexible high frequency coaxial cables. Measurements are performed in the frequency domain from 1 GHz to 9 GHz using 101 frequency points and a total sweep time of 90 ms. Each antenna is mounted to an LES 4 cross table (Isel, Eichenzell, Germany). The transmitting antenna (top) and the receiving antenna (bottom) point to each other and scan the breast in a meander geometry. The cross tables are moved by an iPU-EC servo unit (Isel, Eichenzell, Germany). The whole system is controlled by an iPC25 (Isel, Eichenzell, Germany) using a Matlab interface.

### 2.3. Signal Processing Techniques

In this work, we assume that the upper compression plate is not tilted and bended during compression and maintains parallel with respect to the lower compression plate. This assumption is valid here due to the solid guiding bar on both sides of the compression mechanism. The frequency-domain data measured with the VNA are transformed to the time-domain using an inverse Fourier transform. This leads to the time-domain radar signal s(x,y) measured at position (x,y). A qualitative two-dimensional microwave image I(x,y) can be computed by the root mean square (RMS) according to

(1) $$I\left(x,y\right)=\sqrt{\frac{1}{n}\sum_{i=n_{lL}}^{n_{uL}}s_{i}{\left(x,y\right)}^{2}},$$

where *n* denotes the number of samples in time-domain between a lower bound $n_{lL}$ and an upper bound $n_{uL}$. The lower bound can be defined e.g., by the direct path from the transmitting to the receiving antenna in air. On the other hand, the upper bound depends on the tissue properties. This value must be chosen in such a way that effects related to multipath, e.g., reflections from metallic parts of the measurement setup, should be minimized as much as possible.

### 2.4. Description of the Clinical Work Flow

The clinical work flow is in accordance with the ethical approval and is described in the following: inclusion criteria for a selection of suitable patients were a minimum age of 18 years, occurrence of a tumor in the breast tissue, detected in a routine clinical examination, and oral as well as written informed consent of patients according to the GCP and respective national and international regulations. The patients had undergone a standard X-ray mammography before and afterwards were considered for the proposed examination.

At the beginning, the research physician informed the patient regarding the study, examination procedure and the microwave data acquisition. The patient was asked to undress the upper body prior to the examination. Before starting, a test run of the microwave acquisition system was performed to check the functionality and safety of the system. The patient sat on a height-adjustable chair in front of the measurement system such that the breast was at the same level as the compressing plexiglass plates. The study physician localized the tumor through manual examination. Additionally, the mammography image acquired before was also opened on the clinical workstation, located next to the microwave system, in order to find the tumor position more precisely. The patient placed her tumorous breast between the plates such that the tumor area was located within the 50 mm × 50 mm scan field, which was indicated on the upper plexiglass plate. The size of the scan area was limited by the available time period for the measurements of 3 min. The upper plate was brought down to compress the breast (as it is performed in standard mammography examinations) and was then fixed. After completion of the scan, the contour of the compressed breast was marked on the upper plate for the purpose of comparison and accordance of the acquired data with the mammography image. Finally, the plexiglass plate was lifted, the breast was released and the patient was accompanied to the changing room. The acquired microwave data were saved anonymously on the systems controlling PC.

## 3. Results

**Figure 2:**
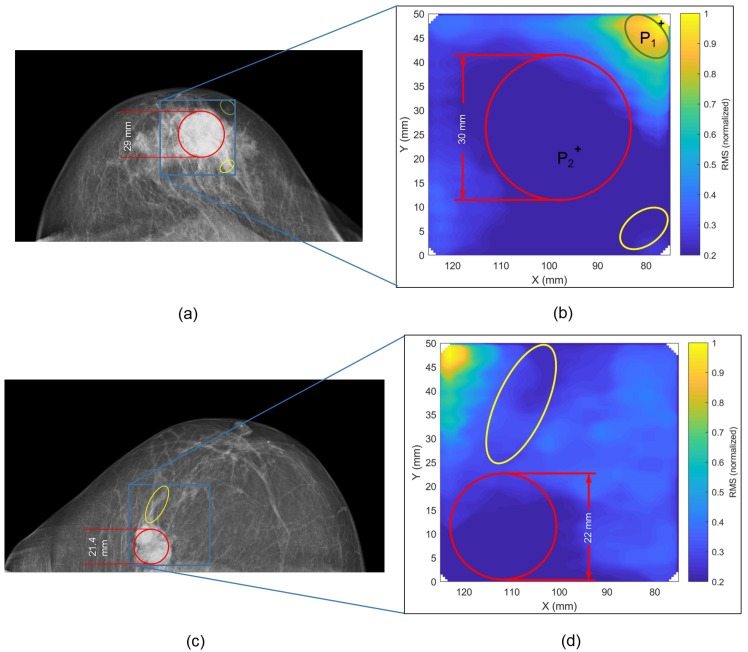
Comparison between X-ray image and microwave image for patient A (**a**,**b**) and patient B (**c**,**d**). The RMS was normalized by the maximum RMS of the scanned region of the present patient. The lowest intensity values occur in the area of the tumor location given by stronger attenuation of cancerous tissue. The final thickness of the compressed breast during microwave examination is very similar for patients A and B, i.e., 4.4 cm for patient A and 4.3 cm for patient B. During X-ray examination, the breast was slightly more compressed, i.e., 4.1 cm for patient A and 3.7 cm for patient B.

Figure 2 shows a comparison between X-ray images and microwave images for two different patients, i.e., patient A (age: 80 years) and patient B (age: 75 years). The blue rectangle in the X-ray images indicates the limited scan area during microwave data acquisition. In both cases, the tumor can be identified on the images of both modalities (red circle) revealed by lower pixel intensities at the tumor location coming from higher signal attenuation of cancerous tissue. In addition, some anatomical features inside the breast next to the tumor can be recognized (see yellow and green ovals in Figure 2a–d).

**Figure 3:**
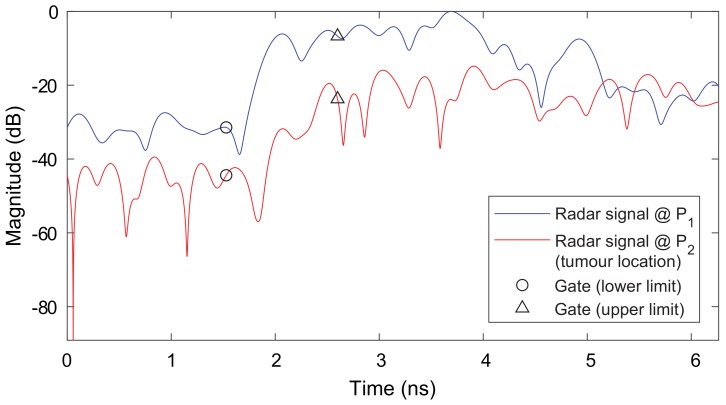
Comparison of UWB radar signals measured at two different positions on the breast as illustrated in Figure 2b. It can be observed that a difference in time of arrival and signal amplitude occurs. Attenuation and time delay are much higher for the tumor location given by a higher conductivity of the malignant tissue and a higher relative permittivity (i.e., smaller wave speed). The time-domain gatings are illustrated that show the signal regions used for RMS computation.

Figure 3 depicts two radar signals in the time-domain that were measured at two different positions on the breast as marked in Figure 2b. One radar signal is measured at the location of the tumor and the other is measured outside the tumor region. Given by the higher relative permittivity and conductivity of malignant tissue, a difference in time of arrival and signal amplitude can be observed. Moreover, this figure illustrates the time-domain gating (characterized by a lower and upper limit) used for the RMS computation in Equation (1).

## 4. Discussion

After successful application of the breast imaging prototype in the laboratory by means of breast phantoms [21], the present work shows first imaging results from breast cancer patients. The clinical measurements represent an important step forward towards the larger acceptance of microwave imaging techniques for medical diagnostics. A major advantage of the proposed system compared to other microwave methodologies is given by simple image formation, which does not require large computational resources. For each measurement, only the inverse Fourier transform must be computed followed by the calculation of the RMS-value for the pixel amplitude at a specific *x*–*y* coordinate. This means that the whole processing chain can be implemented on a microcontroller platform rather than a powerful work station, which is beneficial in terms of reduced system costs and reduced computation time. Portability is an additional feature due its compact size.

The dielectric properties within the breast change locally and beamforming procedures struggle with finding the optimal values for the relative permittivity to realize a proper focussing. Digital beamformers might be improved by studying more complex techniques that take the distribution of relative permittivity into account [22]. In any case, generally the information about the breast’s permittivity is not available on a patient-specific basis and can only be estimated on average [23]. An advantage of the proposed RMS-approach is given by the fact that information about wave propagation inside the breast, e.g., in terms of wave speed, is not required.

Another benefit of the proposed system is the ability to deal with variable breast sizes and shapes by compressing the breast similar to X-ray mammography. This approach avoids inserts that are often used in three-dimensional imaging systems [6] to compensate for variations in breast size. Exploiting the transmission signals through the breast also eliminates the need for surface artifact removal algorithms before the image formation [24]. A drawback of the proposed approach might be given by the compression-induced pain during examination. According to the patients’ feedback, this pain was acceptable.

We are aware that the study has limitations in terms of the number of patients that have been examined. Further limitations are given by the relatively long scan time, which limits the inspection area to about 50 mm by 50 mm. This could be improved by additional engineering that uses multiple transmitters and receivers either in a static or moveable linear array. In that case, the measurement time can be reduced to about 20 s with a full coverage of the breast. Finally, it can be said that the preliminary results shown here are encouraging to study the planar microwave imaging system in more detail in the future. With that being said, we aim at “designing sufficiently powered, large-scale clinical trials to address sensitivity and specificity” [18]. Such clinical trials are important for a statistical analysis to evaluate the MWI systems performance.

## 5. Conclusions

In this paper, a UWB microwave breast imaging prototype system is presented that operates in the frequency range from 1 to 9 GHz. In contrast to many other prototype systems, the proposed approach compresses the breast similar to X-ray mammography. Based on the analysis of the transmission signals, it was possible to identify the tumor by exploiting the stronger attenuation of malignant tissue. It was demonstrated by two patients that breast cancer could be successfully detected. The results were validated by clinical X-ray images.

## Abbreviations

MWI	microwave imaging
RMS	root mean square
UWB	ultra-wideband
